# A collaborative approach to applying Natural Language Processing (NLP) to Domestic Homicide Reviews (DHRs): A study protocol

**DOI:** 10.1371/journal.pone.0348948

**Published:** 2026-05-21

**Authors:** Darren Cook, Elizabeth A. Cook, Sumanta Roy, Ravi Thiara, Rani Selvarajah

**Affiliations:** 1 Violence and Society Centre, City St George’s, University of London, London, United Kingdom; 2 Imkaan, London, United Kingdom; 3 Department of Sociology, University of Warwick, Coventry, United Kingdom; Indian Institute of Information Technology, INDIA

## Abstract

Since 2011, there has been a statutory requirement in England and Wales to conduct a Domestic Homicide Review (DHR) into any domestic abuse-related death: a multi-agency review into the death of a person aged 16 or over that appears resulting from violence, abuse or neglect from an intimate partner, family member or household member. However, analyses of large numbers of DHRs are rare. One of the core challenges is the time and effort required to analyse narrative text within reports. Doing so manually is both time-consuming and resource-intensive and is a primary reason why researchers typically focus on only a portion of the available data. Natural Language Processing (NLP)—a sub-branch of artificial intelligence that enables computers to interpret and process natural language—provides a viable and scalable alternative by offsetting much of the heavy data processing to a computer. This protocol outlines a study to assess the feasibility of applying NLP to DHRs. The study will take a collaborative approach which balances the speed and scale of automation with the embedded knowledge and expertise of practitioners. This approach helps to ensure that outputs of NLP are sensitive and transparent about the biases common within datasets on violence and abuse. Based on initial consultations, we identify the priority research questions for investigation. In addition, we outline details of an ongoing collaboration with one partner, Imkaan. The protocol describes the data access, and retrieval and analysis stages before summarising how feasibility will be evaluated. We anticipate that several challenges will emerge within this study and identify strategies for mitigation. We suggest that working with practitioners who hold deep contextual knowledge about the social realities of violence and abuse, including language, risks, and experiences, mean that tools can be developed that are accountable to communities and appropriately applied to real-world problems.

## Introduction

Domestic abuse (DA) is a widespread public health problem, causing serious harm to victims and their families. Between April 2023 and March 2024, an estimated total of 262 domestic abuse-related deaths were identified by police in England and Wales, including intimate partner and family homicides as well as suicides or unexpected deaths preceded by domestic abuse [1]. Understanding how and why these deaths occur is essential to informing accountability and prevention.

Since 2011, there has been a statutory requirement in England and Wales to conduct a Domestic Homicide Review (DHR) into any potentially DA-related death. A DHR is a multi-agency review of:

the circumstances in which the death of a person aged 16 or over has, or appears to have, resulted from violence, abuse or neglect by a) a person to whom he was related or with whom he was or had been in an intimate personal relationship, or b) a member of the same household as himself, held with a view to identifying the lessons to be learnt from the death. (Domestic Violence, Crime and Victims Act 2004, s9(1))

A review involves individuals that had contact with the victim or perpetrator (e.g., from services, or families and friends) sharing and reviewing information about the circumstances leading up to a death. The stated purpose of a DHR is to learn lessons from the death about how organisations work together to protect victims, how policies and systems might change to prevent deaths in the future, and to “contribute to a better understanding of the nature of domestic violence and abuse” [2, p.6]. Depending on their writing, a DHR can contain rich information on the gendered contexts, motives and circumstances of violence, as well as system changes, contact, and failures. This can include information on the method of killing, the presence of risk factors (e.g., non-fatal strangulation, previous threats to kill), and any history of abuse. A report will typically provide a chronological account of recorded or reported interactions between the victim and perpetrator, as well as their contact with different services (such as health and social care, police, safeguarding units, mental health services, probation etc.) meaning that these documents are often detailed accounts of a victim’s life and death. However, there is no systematic process for the collection or aggregation of data from reviews. Although DHRs are centrally overseen by the Home Office, they are locally commissioned and therefore variable in quality and format. While the launch of a national repository in September 2023 is a promising development, there remains no central system for gathering information from DHRs in a routine or systematic manner. Several jurisdictions have developed national datasets and ‘clearinghouses’ that seek to centralise the death review system and create aggregate data for the purpose of routine analysis of domestic or family violence homicides. Examples include Australia [2], New Zealand [3] and the United States (US) [4]. At present, no equivalent platform exists within England and Wales, although the Welsh Government have recently launched a Single Unified Safeguarding Review (SUSR) that incorporates adult practice reviews, child practice reviews, DHRs, mental health homicide reviews, and offensive weapons homicide reviews [5].

Not only do we lack a systematic means of extracting information from DHRs, but there is also limited oversight of how many reports exist in total. Although estimates suggest approximately 1,200 reports have been commissioned [6], only half are currently available via the Home Office repository. It is therefore important that any information gathering workflow is capable of adapting to an incrementally expanding corpus as new reports are published.

The volume and complexity of the reports prohibit routine, exhaustive analysis through traditional (i.e., manual) efforts. What we *do* know from DHRs is the product of large-scale–but infrequent and sporadic–analyses [7,8], a handful of region-level reviews [9] and academic studies focusing on specific issues (e.g., examining DA-related deaths and cancer [10]). No annual review of the DHR system or newly published reports is conducted (such as those produced by the Child Safeguarding Practice Review Panel [11]).

With the recent launch of the Domestic Abuse Commissioner’s [12] blueprint for an *Oversight Mechanism*, increased focus and support will be placed on local areas developing their data collection mechanisms to track the impact of DHRs. Similar initiatives can be found in the Preventable Deaths Tracker [13] which utilises Prevention of Future Death (PFD) reports. However, PFDs are different review mechanisms, are often very short documents (around 1–4 pages), and can offer only limited insight into domestic abuse. Without routine and systematic data extraction and synthesis of findings from DHRs, it is not possible to identify patterns of violent or abusive behaviour, nor points of intervention or change that can prevent domestic abuse in the future.

One of the core challenges prohibiting more regular large-scale analysis of DHRs is the time and effort required to analyse the text within the reports. Doing so manually is both time-consuming and resource-intensive and is a primary reason why researchers typically focus on only a portion of the available data. Natural language processing (NLP)—a sub-branch of artificial intelligence (AI) that enables computers to understand and process natural language—provides a viable and scalable alternative by offsetting much of the heavy data processing to a computer. Examples of the kinds of tasks where NLP excels include identifying linguistic patterns in unstructured text (e.g., topic modelling), extracting explicit references to people, places, and events (e.g., named entity recognition), and their relationships to one another (e.g., relationship extraction), and classifying the sentiment or emotion of text (e.g., sentiment analysis). NLP methods are becoming increasingly common in public health research due to the ability to efficiently generate analysis of large quantities of documents, reports, and case notes [14]. The ability to analyse entire corpora also provides a means to identifying systemic patterns, measure previously qualitative phenomena, and detect signals not captured in structured data fields.

However, while NLP has been widely adopted across various industries over the last decade, including healthcare, marketing, and finance, its application in areas of violence, abuse and safeguarding policy has received comparatively little attention. Such caution can, in part, be explained by concerns regarding the trustworthiness of automated systems in high-risk areas of public safety. NLP systems are inherently probabilistic — even state-of-the-art models can, and will, make mistakes, which can pose a challenge in settings where tolerance for error is low. Failure to adequately prepare for and manage error in the design and deployment of NLP models can result in biased, and even discriminatory, outcomes, and may lead to risks to public safety, as well as reputational damage for institutions. The use of data in these ways can determine how resources are allocated, inform how law or policy are made, and make decisions about issues that affect everyday life (e.g., assessing risk, detecting abusive behaviours, and providing helpline information for help-seekers) [15]. Therefore, ensuring that tools and technologies are applied in ways that reflect evolving community need is essential.

Taking a collaborative approach to applying AI tools can have significant benefits within the context of domestic abuse. Large amounts of free-text data that contain information relevant to the nature and extent of DA and its prevention are increasingly accessible, though messy and complex [16]. Automating processes of retrieval, sifting, and extraction of these data can bring benefits of scale and speed to a sector which is under-funded and stretched [17,18]. Balancing this automation with the embedded knowledge and expertise of practitioners can ensure that the outputs of NLP are sensitive and transparent about the biases of ethnicity, gender, age, sexuality and disability inherent in that data and the data that such tools are trained upon [19]. Collaboration with voluntary and community sector organisations (VCSOs), survivors and practitioners who hold deep contextual knowledge about the social realities of violence and abuse, including language, risks, and experiences, helps ensure that the tools developed are accountable to communities’ needs and can be applied to real-world problems. This approach also guards against the unnecessary or premature application of NLP, recognising that our results may find current technology unsuitable to adequately analyse the narrative text within DHRs. Such caution is warranted given the costs and risks associated with applying AI in social contexts, which include prediction bias, data scarcity, misrepresentation, privacy concerns, and environmental impacts [20]. In addition, as VCSOs regularly contribute to, conduct and are impacted by the information that DHRs contain, developing NLP tools and applications which speak to the priorities of these organisations and can inform how they make use of DHRs in the future can support equitable and responsible use of AI technology.

## Materials and methods

### Study aims

This protocol outlines a study to assess the feasibility and utility of applying NLP to DHRs in collaboration with Imkaan, a UK-based umbrella organisation committed to addressing violence against Black and minoritised women and girls (VAWG). The study has three key aims:

1. To assess whether NLP can be leveraged to:
  a. Scale analyses of DHRs: previous studies using DHRs have been limited by region or focusing on a specific issue, with large-scale analyses only being conducted at irregular intervals. This study will be the first to utilise the entirety of the DHR Library (publicly and freely available via: https://homicide-review.homeoffice.gov.uk) which, to date, is populated with a total of 611 completed and published DHRs. Conducting such an analysis manually is naturally limited by resource capacity.
  b. Highlight patterns and relationships that have not previously been identified in research: by utilising a larger sample of DHRs for this study, we will assess the feasibility of whether NLP models can be used to identify and interpret complex problems and relationships. Leveraging NLP to identify these relationships before conducting in-depth (human-driven) qualitative analyses to interpret ensures that NLP is used to assist research capacity, rather than replace it.
2. To ensure voluntary and community sector organisation (VCSO) involvement in developing NLP applications and recognising model limitations: different types of data on violence and abuse can hold bias. This can include both how DHRs report demographic data, as well as the nature of the recommendations that they produce. Therefore, it is important that we recognise how NLP might recycle these biases and what processes can be put in place to prevent bias.
3. To identify outputs of high impact/utility to VCSOs impacted by reviews of DA-related deaths: VCSOs are regularly involved in the DHR process and impacted by the recommendations that DHRs produce. Therefore, it is essential that VCSOs are involved in research that makes use of DHRs as well as research that can improve DHRs. Practitioners hold substantive expertise regarding the nature, risks, and dynamics of domestic violence and abuse that can shape the direction of research.

### Identifying research questions

This study is part of a large UK Prevention Research Partnership consortium, VISION (Violence, Health and Society). The consortium is a collaboration of specialised services, criminologists, sociologists, data scientists, economists, epidemiologists, drawing upon a wider network of national and local government departments, police, health trusts, and think tanks.

The first stage of this project consisted of an initial call for collaboration to interested partners. This call was sent to partners who had either previously been involved in a DHR (as a panel member, chair or report author) and/or had offered services in relation to issues typically covered or addressed in a DHR. These requests were sent via email in October 2024, with a further follow-up in January 2025. Interest was received from five individuals/organisations with which follow-up discussions were conducted. This included representatives from the Domestic Abuse Commissioner’s Office and three VCSOs (Imkaan, Women’s Aid, and SafeLives), as well as a member of the National Centre for Violence Against Women and Girls and Public Protection (NCVPP). An initial round of consultations and discussions were held in December 2024 and January 2025 over Microsoft Teams. In these discussions, EC and DC explained the context of the research, the potential role of NLP, and to what extent this technology would be of any utility in applying to DHRs. We asked partners whether there were specific questions or priority areas that it would be useful to explore using NLP in relation to their organisation’s work. We discussed that these applications would be limited by if, and how, these questions firstly, could be answered *within the scope* of a DHR, secondly, were within the scope of a DHR *and reported to sufficient quality*, and thirdly, NLP could feasibly be applied to these ends. We chose to engage a limited number of organisations at this stage to reflect the *depth* of expertise that each service holds in their field, including knowledge representing minoritised and under-represented voices in the DA sector. Therefore, we did not intend for this consultation to be representative, but a purposeful selection of expertise. We also supplemented by reflecting on our ongoing qualitative analysis of DHRs as well as evidence briefings completed commissioned by the Domestic Abuse Commissioner’s Office [21].

From these discussions, a draft list of potential research questions was generated which were then ranked based on two factors: impact and feasibility. Impact was assessed based on the usefulness to the partner based on previous discussions and whether existing data could be identified elsewhere that could be employed to answer the question. Feasibility was assessed based on both the predicted capability of NLP, complexity of the research question, and the availability and quality of free-text available within a DHR. The results of this exercise are summarised Table 1 below.

**Table 1:** Longlisted research questions identified through first phase of collaboration.

#	Research question	Impact	Feasibility
1	What was the level of involvement of by and for organisations or Black and minoritised/ ‘cultural experts’ experts or as ‘Chairs’ in DHR panels?	High	Feasible
2	What was the housing status or housing issues identified in DHR reports involving Black and minoritised women?	High	Feasible
3	What is the demographic breakdown – gender, ethnicity, immigration status, other protected characteristics – within DHR reports?	High	Feasible
4	How many victims were known to/ had contact with agencies prior to their death and who were they (e.g., specialist support)? How does this compare across group, e.g., for Black and minoritised women?	High	Feasible
5	How many were not known to any agency (the demographic breakdown) and migrant status and region / Local Authority Area?	High	Challenging
6	How many times are ‘lack of follow-up’ and ‘agency failure’ mentioned?	Medium	Feasible
7	How many times is ‘victim disengaged from support’ mentioned?	Medium	Feasible
8	What is the extent to which ‘cultural training’ or ‘cultural competency’ or diversity training is mentioned in the learning / recommendations / which agencies are recommended for training?	Medium	Feasible
9	To what extent is racialised victim-blaming language used to describe Black and minoritised victims of domestic homicide? E.g. ‘difficult’, ‘challenging’, ‘hard to reach’, etc.	Medium	Feasible
10	Are language barriers referenced in interactions with statutory services and was an interpreter offered?	Medium	Feasible
11	How do profiles of domestic homicide compare to domestic abuse-related suicide (specifically, intimate partner homicides) (e.g., victim/perpetrator and incident characteristics, type of abuse, risk factors, service contact/engagement)?	High	Feasible
12	How many times are references to self-harm, suicidality, and suicide attempts mentioned in DHRs?	High	Feasible
13	Do Chairs discuss the impacts of abuse on the victim?	Low	Challenging
14	For those identified as a primary abuser in the domestic homicide, and where there is mention of having previously experienced an incident of domestic abuse (e.g., police being called out to a domestic abuse incident where the domestic homicide victim is identified/labelled as the perpetrator), were these single incidents or a series of incidents? How were they identified and were they misidentified?	Low	Challenging
15	What attempts are made by professionals (i.e., midwives, social services) to ask about domestic abuse without the associated perpetrator present? Was there a vulnerability or support need, i.e., language barrier?	Medium	Challenging
16	When disclosures of domestic abuse were made to statutory agencies, were referrals made to domestic abuse services?	Medium	Feasible
17	In how many DHRs were there child contact arrangements in place?	Medium	Feasible
18	How many DHRs explicitly identify honour-based abuse?	Medium	Feasible
19	In how many DHRs was a DASH assessment carried out and what was the outcome?	Medium	Feasible
20	In how many DHRs was a MARAC conducted and what was the outcome?	Medium	Feasible
21	Where Section 7 requests are made, are welfare reports conducted?	Not sure	Feasible
22	How are pseudonyms used? How are relationships of dependency created between individuals?	Low	Unfeasible
23	In cases of separation, has child contact been assessed as being a risk for domestic homicide or suicide (and whether social services, police, etc. report it as a risk)?	Medium	Feasible
24	How are DHR recommendations clustered across child/adult social care, health, criminal justice sectors?	High	Feasible
25	How is victim-blaming language present and used in DHRs?	Medium	Feasible
26	How does intersectionality shape help-seeking, types/experiences of abuse, needs, referral pathways / services in contact with (including whether in contact with specific DA services around their identity), length of abuse, risk assessment and source (rating and differences by referral route)?	High	Feasible
27	What are the facilitators to help-seeking?	Medium	Feasible
28	What organisations took which actions, e.g., direct referral, or instructed them to self-refer?	Low	Challenging

**Fig 1:**
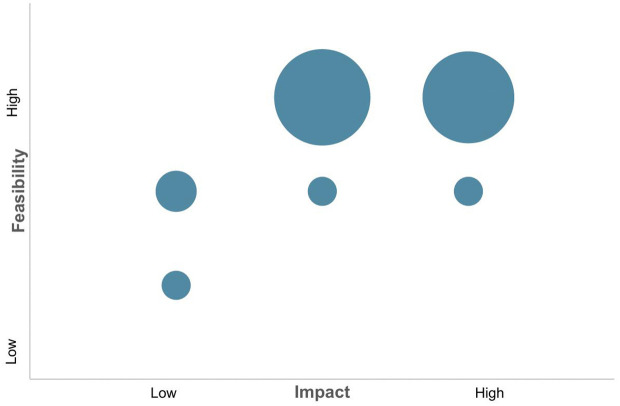
Proportion of research questions by feasibility and impact here.

The initial phase of the collaboration generated a draft list of 28 viable research questions addressing both the nature and extent of different types of domestic abuse (for example, types of abuse experienced by different demographic groups), as well as the procedural aspects of how DHRs conducted (for example, the involvement of different panel members or the use of victim-blaming language). These are represented visually in Fig 1 below.

Accounting for impact and feasibility, we created a finalised list of research questions that was thematically synthesised into five priority areas. These priorities are summarised in Table 2 below.

**Table 2:** Shortlisted research questions synthesised by theme.

#	Priority area	Research questions
1	Quality and completeness of victim and perpetrator demographics	How are victim and perpetrator demographic characteristics recorded? For example: age; sex; gender identity; ethnicity; migrant status; disability; religion; sexual orientation; financial context (receipt of welfare support); housing status.
2	Extent of statutory and non-statutory service involvement prior to a death	What is the extent of service involvement (or lack thereof) prior to a DA-related death?
		How many were not known to any agency (or had minimal contact) including by demographic (e.g., how many Black and minoritised women were not known to statutory agencies)?
		Types of referral pathways and help-seeking patterns?
		What are the facilitators and barriers to help-seeking?
3	Victim-blaming language	To what extent is victim blaming language used to describe victims and does this differ by demographic?
		To what extent is language such as ‘difficult’, ‘challenging’, ‘hard-to-reach’, ‘victim disengaged’ used?
4	Comparing DA-related homicides to other DA-related deaths (e.g., suicides)	How do domestic homicides compare to other domestic abuse-related deaths (specifically, intimate partner homicides) (e.g., victim/perpetrator and incident characteristics, type of abuse, risk factors, service contact/engagement)?
		To what extent have self-harm, suicidality and suicide attempts been reported?
5	Clusters of DHR recommendations	How are recommendations clustered across child/adult social care, health, and criminal justice in response to specific types of death?

This phase of the collaboration generated five priority areas that will guide this feasibility assessment of applying NLP to DHRs. Firstly, we will apply NLP to identify the availability of information that is reported on victim and perpetrator demographics within DHRs, as well as assess the quality of how different demographics are reported. Secondly, we will identify the extent to which different services were involved prior to a death, such as police, VCSOs (including specialist ‘by and for’ organisations which are “run by and for the users and communities they aim to serve” [22, p.2]), schools, health care, social care, and employers etc. We will also assess whether service involvement differs by victim or perpetrator group. Thirdly, we assess whether NLP can be applied to identify and interpret victim-blaming language, including whether this language changes according to different victims and perpetrators. Fourth, we will compare characteristics of domestic homicides (specifically, intimate partner homicides) to other DA-related deaths to understand how they differ in terms of victim and perpetrator demographics, nature and extent of abuse, and type of service involvement. Recent qualitative analyses of DA-related suicides have demonstrated that this is an emerging but under-recognised area of research and, as yet, have not been compared to domestic homicides [17,23]. Fifth, we will assess the feasibility of using NLP to identify clusters of recommendations made to different services. For example, whether types of recommendation (e.g., additional training, education) are associated with certain services or sectors (e.g., adult or child social care, police, health). This collaborative approach to generating research questions therefore ensures that outputs address both substantive and procedural issues to improve the conduct of DHRs as well as the knowledge that they produce.

### Study setting and design

There has been a statutory requirement in England and Wales to conduct a DHR into any DA-related death since 2011, with a central repository collecting these reports available since November 2023. The study setting for this project will therefore be restricted to England and Wales, acknowledging that models of DA-related death reviews are either recently established or currently under development in other jurisdictions.

### Data source

DHRs are statutory multi-agency reviews that aim to identify lessons that can improve responses to domestic abuse and prevent future deaths. Each review involves analysing information from a range of agencies and practitioners. A DHR Chair will also seek information from family members and the wider community. A core purpose of a DHR is to build a picture of the circumstances leading up to the victim’s death.

While the format and quality of DHRs vary due to local commissioning practices, all include detailed free-text narratives, chronologies, and agency analyses. Reviews vary in length, often spanning 30–150 pages These documents contain rich qualitative data on victim-perpetrator dynamics, service contact, missed opportunities for intervention, and policy or system-level recommendations for change.

### Data acquisition

Reviews will be accessed and retrieved from the Home Office DHR Library using an automated web scraper developed in Python by one of the authors (DC). As part of responsible data collection, the library website has been checked for a robots.txt file, a standard web protocol used to indicate whether automated tools such as web scrapers are permitted to access site content. At the time of writing, no such file is present on the DHR Library website, suggesting there are no stated restrictions on automated access. Once a review URL is identified by the scraper, the corresponding PDF will be downloaded and processed by a secondary function that extracts the full document content and saves it locally for further processing. All publicly available DHRs in the Library will be included at the outset, with no additional inclusion or exclusion criteria applied at this stage.

### Data cleaning and preparation

The text of each PDF will be parsed using a dedicated Python script. As PDFs are not inherently machine-readable, several automated preprocessing steps will be applied to improve the quality and consistency of the extracted text. These include removing boilerplate content such as headers, footers, footnotes, page numbers, title pages, URLs, and tables of contents. Where possible, headings and subheadings will be inferred based on font size and formatting features. Any tables present in the documents will be either standardised into plain-text format or removed, depending on their structure and extractability. The output of each preprocessing step will be subject to spot-checking and revision to ensure a high level of data extraction.

### Natural language processing

The first step in NLP will be to transform the text into a format a computer can understand. This involves preprocessing steps such as tokenization, part-of-speech tagging, stemming, and dependency parsing [24]. Unlike humans, machines require text to be represented numerically rather than linguistically. Common machine-readable ways of representing text include word frequencies (the count of a word in a document) or embeddings — high-dimensional vectors that capture the semantic meaning of a word based on its context within a larger corpus [25]. Projecting text into a high-dimensional vector space enables us to perform common NLP tasks including classification, clustering, and information extraction. The precise techniques we will use will differ between each of the priority areas and research questions outlined in Table 2.

1. *Quality and completeness of reporting of victim and perpetrator demographics* — This analysis will involve collating and tabulating key demographic descriptors such as age, ethnicity, and gender. Recording of this information is not standardised within DHRs, based on a preliminary review, and is instead inconsistently referenced under headings such as ‘Background’, ‘Family Background’, or ‘Chronology’. We will develop a combination of keywords and syntax rules to determine if a specific text segment contains demographic information. If necessary, we will progress to more sophisticated semantic information retrieval methods to capture more nuanced or implicit references to demographic information. Descriptive statistics will then assess the frequency and completeness of demographic reporting.
2. *Extent of statutory and non-statutory service involvement prior to a death* — The vast majority of DHRs *should* contain a chronology of events, outlining the activities that took place prior to the victim’s death. These chronologies present information in temporal order, enabling a systematic extraction of the relevant events. We will identify and extract references to both statutory and non-statutory service involvement using a combination of rule-based matching, named entity recognition (NER), and event detection techniques. These methods enable us to identify points of service contact even when phrased inconsistently or indirectly. Extracted data will then be used to analyse the frequency, nature, and timing of service involvement prior to death.
3. *Victim-blaming language* — We will develop and validate a victim-blaming lexicon based on the surrounding literature on DA discourse. This lexicon will include words, phrases, and rhetorical constructions associated with the attribution of blame or responsibility to victims. We will then apply this lexicon to each DHR to assess the prevalence of victim-blaming, using frequency-based and context-aware techniques. We can also examine variation in the use of victim-blaming language across demographic groups by linking this analysis to the demographic detection process described in priority area #1.
4. *Comparing domestic homicides to other DA-related deaths* — Using linguistic markers and content cues, we will classify DHRs into subgroups of interest, including IPV-related, suicides following abuse, and other DA-related deaths. Classification will be carried out using a combination of rule-based keyword matching, and document-level text classification approaches. For the latter, we will explore few-shot learning techniques (such as those described by Brown et al. [26]) to support accurate classification using minimal annotated training data. Once categorised, we will use information extraction techniques to compare incident characteristics, patterns of abuse, and risk factors across these subgroups.
5. *Clusters of DHR recommendations* – Each DHR typically concludes with a series of recommendations, which can include general recommendations as well as agency-specific guidance. We will use a combination of unsupervised machine learning techniques including Latent Dirichlet Allocation (LDA) [27] to identify recurring themes and patterns in recommendations. To facilitate a more fine-grained sectoral analysis, we will use the embedded agency subheadings to group recommendations by originating organisation (e.g., police, health, social care). This will enable us to examine both global thematic trends as well as sector-specific patterns in post-incident learning.

All code produced for this project will be collated, stored, and publicly accessible via GitHub under an MIT licence (allowing others to use, modify, and redistribute the code without seeking formal permission). The repository will include detailed documentation in the form of a README markdown file, as well as demonstrations via annotated notebooks and example workflows to encourage reuse and reproducibility.

### Evaluation and feasibility criteria

Evaluating the components in an NLP information extraction system requires a combination of quantitative and qualitative performance metrics. We define a set of feasibility criteria to avoid post-hoc rationalisation and ensure a high-level of transparency, based on coverage, extractability, model performance, and relevance to stakeholders. *Coverage* metrics assess completeness, and whether the proposed methodology captures the relevant concepts in full. *Extractability* covers how accurately relevant information is identified via precision and recall metrics, while m*odel performance* is used to evaluate how well a technique accomplishes its stated task using global measures such as accuracy and F1 scores. Lastly, *stakeholder interpretability* assesses the relevance, utility, and informativeness of the output in a practical context. Collectively, these measures allow us to rigorously evaluate whether NLP can be reliably, responsibly, and meaningfully applied to DHRs. These criteria are summarised in Table 3 below.

**Table 3:** Evaluation plan split by thematic area.

#	Thematic Area	Coverage	Extractability	Model Performance	Stakeholder Interpretability
**1**	Victim & perpetrator demographics	% of DHRs containing basic demographic information	Precision / Recall scores of extraction techniques.	Manual validation of entity extraction; F1 score as an overall measure of model performance	Do demographic trends highlight gaps or patterns in reporting practices?
**2**	Service involvement	Proportion of DHRs with structured chronologies	% of successfully identified service contact-points based on a manually labelled subsample	Accuracy of NER for detecting service agency names	Do findings make it clear who was involved, and is the information actionable?
**3**	Victim-blaming language	Presence of victim-blaming language across the corpus	Match rate using lexicon and contextual filtering (e.g., negation detection and semantic nuance).	Precision / recall of victim-blaming detection using an annotated subset; interrater agreement (Kappa) scores between auto and manual annotation.	Do examples adequately capture victim-blaming language? Can stakeholders identify common trends across demographic groups?
**4**	Comparing incident types	Proportion of DHRs clearly indicating the type of incident (e.g., IPV, suicide, other DA).	% of correctly classified DHRs by incident type.	Accuracy / F1 scores of document-level classification models (e.g., comparison between rule-based, few-shot, traditional ML).	Are distinctions between cases meaningful for shaping interventions?
**5**	Clusters of recommendations	Proportion of DHRs with structured recommendations linked to identifiable sectors / agencies	Number of recommendations successfully parsed and linked to agencies; the ability to detect recurring themes	Topic coherence scores (e.g., CV coherence for LDA topics); human validation of clusters (with reported label agreement)	Are recommendation clusters understandable and aligned with stakeholder needs?

### Collaboration

We will take an iterative approach to the analysis and interpretation of the DHRs. Building on the formative consultation work, we will continue to update all stakeholders by sharing findings. In particular, we will work closely with Imkaan to iteratively develop analysis, gather feedback, and ensure that interpretations remain grounded in the lived realities of domestic abuse and service provision. Imkaan have two decades of experience working with other ‘by and for’ organisations to address VAWG, including domestic and honour-related abuse. This collaboration also helps to ensure that inputs to and directions of analyses are supervised by expert stakeholders and feedback loops are built in to avoid amplifying bias that may be found within DHRs (see ‘Discussion’).

Imkaan will be invited to continue to shape the framing of the research questions, reflect on emerging themes, and identify areas requiring further exploration or clarification. This process will help to contextualise the findings and support the development of outputs that are meaningful to both practice and policy. Engagement with practitioners in this sense is to increase the relevance and utility of the research questions and contribute to the wider sector, as endorsed by the Women’s Aid’s *Research Integrity Framework on Domestic Violence and Abuse* [28]. This also means being transparent about who and how you have engaged with partners and acknowledging how the research team shapes research priorities.

Engagement will be adapted to stakeholders’ capacity and preferences, using flexible formats such as virtual meetings, written summaries, or interactive demonstration sessions. Updates will be provided monthly. Outputs and authorship will be agreed upon with stakeholders. Any draft written materials for publication will be provided to relevant stakeholders for review at least three weeks in advance of target submission date.

### Ethical considerations

Although the data used in this study is publicly available, several ethical considerations require careful attention due to the sensitive and potentially distressing nature of the content. DHRs often contain detailed accounts of violence, abuse, and system-level failures, and may refer to individuals, events, or circumstances that are identifiable to those familiar with the case.

To mitigate risks of harm or unintended disclosure, all data processing will be conducted with a strong emphasis on confidentiality and data minimisation. While full names and identifiers are typically redacted in published DHRs, the potential for re-identification, particularly in small or specific communities, will be acknowledged and managed during analysis and dissemination. Extracted data will be stored securely on encrypted, access-controlled university servers in accordance with institutional data protection policies and the UK General Data Protection Regulation (GDPR). Data will be reported at an aggregate level. Any human labelling in support of machine learning classification will use neutral, non-harmful codes, and will utilise a consensus-coding approach (i.e., multiple coders with reported interrater agreement scores). Any outputs produced by an unsupervised machine learning algorithm will be qualitatively reviewed to ensure identified topics or clusters are not stigmatising or harmful. Human oversight at key decision points will be maintained to contextualise ML findings and avoid presenting false narratives. Any qualitative excerpts that are reported as examples will be anonymised and not be identifiable at case level.

This study has received ethical approval from City St George, University of London’s Research Ethics Committee (ETH2526−0304).

### Data governance

All data collection, storage, and processing activities in this study are governed by principles of secure, ethical, and legally compliant data handling. While detailed procedures are outlined in the relevant sections above, data governance is informed by City St George’s policies on research data management and aligns with UK GDPR and the ethical conditions approved under the VISION consortium. One of the authors (DC) has completed the Safe Researcher Training programme offered by the UK Data Service, which provides guidance on best practices for working with sensitive and potentially disclosive information. Data access will be restricted to authorised members of the research team, and all files will be stored on encrypted, access-controlled servers with multi-factor authentication (MFA). No attempts will be made to re-identify individuals, and data will be retained for the period specified in institutional policy (ten years).

### Anticipated outputs and dissemination

This project requires iterative supervision and feedback. Therefore, outputs will be developed in discussion with Imkaan and take into consideration preferred timelines, formats, co-authorship and framing. We anticipate that we will produce manuscripts to be submitted to peer-reviewed journals (prioritising journals that have open-access agreements) and policy briefings available on the project website (vision.ac.uk). Additional outputs will be agreed according to stakeholder interest and capacity. We will also present findings to relevant national and international conferences and meetings. Outputs will also be delivered to relevant research and practice networks (for example, the International Community of Practice on Domestic Homicide) as well as policy units (for example, Home Office Interpersonal Abuse Unit).

Outputs and authorship will be agreed upon with stakeholders in advance. Any draft written materials for publication will be provided to relevant stakeholders for review at least three weeks in advance of the target submission date. The anticipated timeline for this study is outlined in Table 4 below. The first stage (stakeholder engagement) has been completed. The first stage (stakeholder engagement) has been completed. Participant recruitment and data collection are not applicable to this study.

**Table 4:** Study timeline.

Milestone	Activities	Timeline	Outputs
Stakeholder engagement	Initial partner call Consultation Research question feasibility assessment	Feb 25 – Aug 25	Shortlist of research questions
Data acquisition	Automated web scraping Extract text from PDFs	Oct 25 – Nov 25	Corpus of DHRs (stored as plain text files)
Data cleaning	Pre-processing and cleaning Extract structured information from text files	Nov 25 – Dec 25	Creation of a machine-readable dataset
NLP analysis	Analysis of: Demographics Service involvement Victim-blaming language Incident classification Recommendation clustering	Jan 26 – Mar 26	Analytical outputs
Evaluation	Apply feasibility criteria	Mar 26 – Apr 26	Feasibility documentation
Stakeholder iteration	Monthly updates Workshops with Imkaan and partners	Throughout	Iteratively refined analysis
Dissemination	Manuscripts Policy briefings Conference presentations	Apr 26 – Aug 26	Publications and practice outputs

## Discussion

### Feasibility and anticipated challenges in using DHRs

This study will assess the feasibility of applying NLP techniques to the entire corpus of DHRs available via the Home Office repository. While advances in NLP have made large-scale text analysis more accessible, we acknowledge several anticipated challenges.

First, DHRs are variable in their structure, format, and writing style, as they are locally commissioned and authored by different review Chairs and involve different panel membership. Typically, each review consists of an executive summary, individual management reviews (IMRs) for each contributing service, an overview report, and an action plan. The suggested format of each review is recommended within Home Office statutory guidance [29], although variations still remain by report (for example, the inclusion of different subheadings, sections and information). In addition, DHRs address different types of DA-related deaths and victim/perpetrator relationships. Research shows that the gendered nature of DA shifts depending on the *type* of DA. For example, Hoeger et al. [1, p.23] identified that differences in victim sex are more pronounced for deaths in the context of intimate partner relationships (83% female victims), than for familial relationships (55% female victims). These gendered contexts can be captured within a DHR report and are important to distinguish between. These heterogeneities and inconsistencies introduce complexity in designing preprocessing pipelines and in applying models that assume uniform input.

Second, while the text volume is large in aggregate, the total number of reports presents limitations for some advanced machine learning methods (i.e., those based on deep learning architectures), particularly those reliant on very large training datasets. In line with the principle of methodological parsimony, we will prioritise simpler interpretable models (e.g., rule-based, traditional NLP pipelines) over more sophisticated deep learning approaches (e.g., neural networks), unless warranted by the complexity of the analysis or the structure of the data.

Third, ensuring that the outputs of NLP models are interpretable and relevant to expert stakeholders requires careful design of both the modelling approach and the reporting format. We conducted an initial consultation with five organisations and will ensure that they are provided with information at regular intervals. In addition, we will work closely with Imkaan so that decisions are staged sufficiently, and flexibility is built into the timeline and communication formats for outputs.

Lastly, we must take steps to ensure that an absence of a variable of interest within a report is not treated or interpreted as an absence of the characteristic itself. In other words, our approach is intended to evaluate the recording practices (e.g., inclusion, omission and patterning) of DHRs rather than the underlying prevalence of demographic characteristics.

### Bias in machine learning

Bias in machine learning models is a well-documented issue, particularly when applied to sensitive social data and in the public sector [30]. As part of a partnership between the Sexual Violence Research Initiative (SVRI) and The MERL Tech Initiative (MTI), Amazon-Brown et al. [19] developed a guidance resource for researchers engaging with AI in relation to VAWG. Critically, they point out that “human expertise is irreplaceable. AI can improve efficiency, but final analysis and interpretation should always lie with researchers” [20, p.2]. Integrating AI into VAWG research in a controlled and transparent way can is essential to not only minimising inaccuracies but building capacity to use AI responsibly.

In the context of DHRs, model outputs may reflect or amplify biases present in the source documents such as under-reporting of certain identities, cultural and racial stereotypes, or omissions of certain cases altogether [17]. For example, Chantler et al.’s [9, p.487] analysis of 141 DHRs showed that information on the ethnicity was available for just over half of victims and perpetrators, while information on age was missing for just under a quarter of victims and a third of perpetrators. In addition, as no information is available from CSPs on decisions whether to commission cases as DHRs, it is difficult to identify whether certain deaths are being excluded from this dataset [31]. There is also a risk that NLP methods may flatten nuance or obscure contextual detail, particularly if models are trained or interpreted without sufficient stakeholder involvement. To mitigate against this, our study will adopt an iterative and collaborative approach to analysis, ensuring that expert stakeholders are involved in reviewing intermediate outputs and shaping final interpretations.

A related challenge concerns the interpretability of NLP outputs to non-technical audiences. VCSOs, policymakers, and practitioners may not be familiar with the technical language or statistical reasoning behind NLP models, and there is a risk of either over-trusting or underestimating model results. To address this, outputs will be developed in plain language, and visual summaries and contextual explanations will be provided where appropriate. We will also hold demonstration sessions where appropriate and required. Emphasis will be placed on transparency and explainability, including the limitations of the models, the confidence of findings, and any underlying assumptions or preprocessing decisions that affect interpretation.

### Contribution and potential impact

This study is uniquely situated at the intersection of data science and the DA field, with empirical, methodological and practical implications for both.

The study will assess the feasibility and utility of applying NLP to free-text data contained within DHRs. As many studies are often based on a sub-sample of DHRs, and large-scale analyses are limited by resource and capacity, this study will assess whether NLP can meaningfully identify and extract summary data from DHRs as well as understanding its capabilities for interpreting more complex relationships and causal pathways. To date, no study has been conducted utilising the entire corpus of DHRs available within the Home Office library. Therefore, this study has the potential to impact and develop the existing evidence base on DA, in particular, unpicking emerging trends and patterns through large-scale comparisons and interpreting associations between more latent or hidden issues.

This study will also provide a statement on the quality of DHRs as a mode of data collection for the prevention of DA. For example, we will apply NLP to identify the percentage of DHRs that report adequate data on victim, perpetrator, and case demographics (such as age, ethnicity or disability). We will use these findings to make recommendations to improve the conduct, quality and reporting of DHRs. For example, if NLP can assess levels of missing data or interpret differences between DHRs that are conducted in different ways (e.g., where there is family engagement), then guidance could be issued which evidences how DHRs are conducted and reported affects the quality of evidence. Improving the quality of DHRs has the potential to impact organisational learning, as well as identifying the conditions necessary (e.g., technical, structural, resource or otherwise) for developing DHRs as a source of knowledge for practitioners to utilise.

We will demonstrate the importance of ensuring that applications of NLP, including setting the scope, iteratively developing the models, and supervising and checking its results, are generated in collaboration with expert stakeholders. In this study, we will work directly with Imkaan who are involved in DHRs as panel members, are impacted by the recommendations that DHRs generate, and have extensive knowledge about DA and how services operate in response. This has potential for sector-wide impact in two ways: firstly, in improving the quality of reporting in DHRs and therefore the recommendations that they produce and, secondly, in supporting the identification and access to relevant evidence for organisations to conduct their own in-depth research upon.

Collectively, the five priority areas identified through the consultation demonstrate how NLP can support both substantive and procedural insights from DHRs. Analyses of demographic reporting and service involvement provide evidence on the completeness and distribution of key case information, enabling assessment of data quality and gaps in institutional knowledge. Examination of victim-blaming language offers a systematic means of identifying how victims are framed within DHR narratives, including whether such framings vary across demographic groups, with implications for guidance, training, and the interpretation of findings. Comparative analysis of different types of DA-related deaths supports a more nuanced understanding of pathways to fatal outcomes, while clustering of recommendations enables the identification of recurring patterns in organisational learning across sectors. These outputs move beyond descriptive analysis to provide a structured evidence base that can inform improvements in how DHRs are conducted, interpreted, and used to support prevention and policy development.

### Conclusion

As the use of AI accelerates across a range of sectors, a distinct set of challenges emerge for the DA field [19]. Collaborating with expert stakeholders to undertake this feasibility study of applying NLP to DHRs means that we can better understand the benefits and limitations of these types of technologies. We hope this provides not only a technical assessment of whether NLP can be applied to DHRs but contributes to a collective discussion about how to make AI work meaningfully for the public sector. In doing so, this study provides a reflection on the methodological and ethical challenges of using AI in the context of DA and identify best practices for applying NLP in the future
